# Functional and molecular defects of hiPSC-derived neurons from patients with ATM deficiency

**DOI:** 10.1038/cddis.2014.310

**Published:** 2014-07-17

**Authors:** L Carlessi, E Fusar Poli, G Bechi, M Mantegazza, B Pascucci, L Narciso, E Dogliotti, C Sala, C Verpelli, D Lecis, D Delia

**Affiliations:** 1 Department of Experimental Oncology, Fondazione IRCCS Istituto Nazionale dei Tumori, Via Amadeo 42, 20133 Milano, Italy; 2 Department of Neurophysiopathology, Fondazione IRCCS Istituto Neurologico Carlo Besta, Via Amadeo 42, 20133 Milano, Italy; 3 Institute of Molecular and Cellular Pharmacology (IPMC) CNRS UMR7275 and University of Nice-Sophia Antipolis, 660 Route des Lucioles, 06560 Valbonne, France; 4 CNR Institute of Crystallography, Via Salaria, Km. 29.300, 00016 Monterotondo Scalo, Roma, Italy; 5 Department of Food Safety and Veterinary Public Health, Istituto Superiore di Sanità, Viale Regina Elena 299, 00161 Roma, Italy; 6 Department of Environment and Primary Prevention, Istituto Superiore di Sanità, Viale Regina Elena 299, 00161 Roma, Italy; 7 CNR Institute of Neuroscience and Department of Biotechnology and Translational Medicine, Via Vanvitelli 32, 20129 Milano, Italy

## Abstract

Loss of ataxia telangiectasia mutated (ATM) kinase, a key factor of the DNA damage response (DDR) pathway, causes the cancer predisposing and neurodegenerative syndrome ataxia-telangiectasia (A-T). To investigate the mechanisms of neurodegeneration, we have reprogrammed fibroblasts from ATM-null A-T patients and normal controls to pluripotency (human-induced pluripotent stem cells), and derived from these neural precursor cells able to terminally differentiate into post-mitotic neurons positive to >90% for *β*-tubulin III+/microtubule-associated protein 2+. We show that A-T neurons display similar voltage-gated potassium and sodium currents and discharges of action potentials as control neurons, but defective expression of the maturation and synaptic markers SCG10, SYP and PSD95 (postsynaptic density protein 95). A-T neurons exhibited defective repair of DNA double-strand breaks (DSBs) and repressed phosphorylation of ATM substrates (e.g., *γ*H2AX, Smc1-S966, Kap1-S824, Chk2-T68, p53-S15), but normal repair of single-strand breaks, and normal short- and long-patch base excision repair activities. Moreover, A-T neurons were resistant to apoptosis induced by the genotoxic agents camptothecin and trabectedin, but as sensitive as controls to the oxidative agents. Most notably, A-T neurons exhibited abnormal accumulation of topoisomerase 1-DNA covalent complexes (Top1-ccs). These findings reveal that ATM deficiency impairs neuronal maturation, suppresses the response and repair of DNA DSBs, and enhances Top1-cc accumulation. Top1-cc could be a risk factor for neurodegeneration as they may interfere with transcription elongation and promote transcriptional decline.

Ataxia-telangiectasia (A-T) is a pleiotropic disorder with predisposition to cancer and early onset neurodegeneration as key features.^1, 2, 3^ The neuropathological abnormalities in A-T include progressive loss of cerebellar Purkinje and granule neurons, less pronounced degeneration of the bulbar olivae in the brainstem and mild loss of myelinated fibers in corticospinal and spinocerebellar tracts.^4^ A-T is caused by germline mutations inactivating the ataxia telangiectasia mutated (ATM) protein kinase, which has an apical role in the DNA damage response (DDR) to double-strand breaks (DSBs). Upon activation by DSBs, ATM phosphorylates a plethora of substrates implicated in cell cycle arrest, DNA processing and repair, chromatin remodeling, transcription and apoptosis.^1^ Concordant with the role of ATM in the DDR, the neurodegenerative phenotype in A-T has been attributed to a defective DDR in pre- and post-mitotic neurons and ensuing accumulation of genotoxic lesions. However, the fact that cells from A-T patients and specimens from ATM knock-out mice exhibit increased signatures of oxidative stress,^5, 6^ together with the discovery that ATM senses and responds to reactive oxygen species (ROS) through formation of active, disulfide-cross-linked ATM dimers^7^ suggest that the loss of redox balance may contribute to neurodegeneration in A-T.

In neurons, a fraction of ATM is localized in the cytoplasm where it phosphorylates the synaptic proteins VAMP2 and synapsin-1.^8^ Recently, ATM has been detected in dendrites and dendritic spines, and its activation by neuronal firing and synaptic activity induces the phosphorylation of proteins essential for neuronal function.^9^

Neurodegeneration in A-T has additionally been linked to epigenetic modifications associated with deregulation of class II histone deacetylase HDAC4^10^ and hyperexpression of the histone-lysine *N*-methyltransferase EZH2,^11^ altogether inducing transcriptional repression of multiple neuronal genes and cell death.

It should be noted that germline mutations in *ATM* severely impair glial cell functionality and vascular integrity, suggesting that Purkinje cell death and cerebellar degeneration in A-T may result from a dysfunctional neuro-astro-vascular unit.^12^

In summary, several factors are implicated in the neurodegenerative phenotype of A-T, but which of them has the most crucial role is still unresolved, primarily because of the unavailability of model systems able to recapitulate the neurological disease. In this regard, ATM knockout mice do not manifest the progressive ataxia seen in A-T nor the loss of cerebellar Purkinje cells.^13^

The reprogramming of human somatic cells into induced pluripotent stem cells (human-induced pluripotent stem cell (hiPSCs)) by the introduction of pluripotency factors (Oct4/Klf4/Sox2/cMyc) represents a prominent advance in stem cell biology, owing to the capacity of these cells to differentiate to virtually any cell type of the human body, and the possibility to model patient- and allele-specific genetic diseases.^14^ In the case of A-T, the establishment of hiPSCs and conversion to functional neurons has been recently reported.^15, 16, 17^

In this study, we performed a previously undocumented functional and molecular analysis of hiPSC-derived A-T neuronal cells harboring patient-specific mutations, in order to shed light on the causes of the neuropathology in A-T.

## Results

### Generation and characterization of hiPSCs derived from A-T and Ctrl fibroblasts

**Figure 1:**
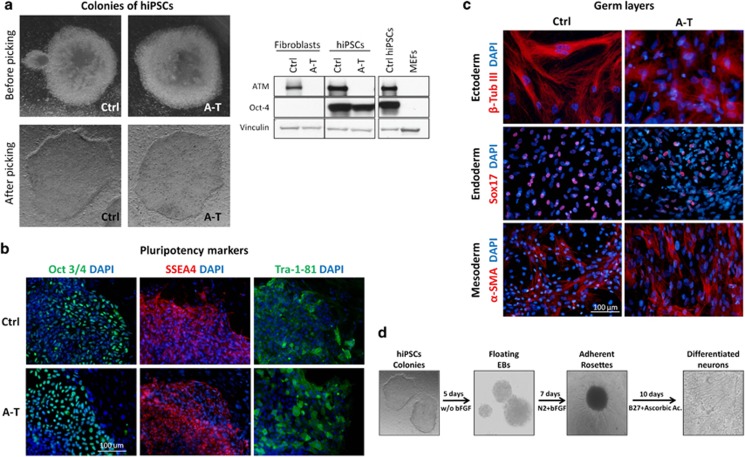
Generation, characterization, pluripotency validation and neuronal differentiation of hiPSC colonies from Ctrl and A-T patients. Representative images of newly formed hiPSC colonies before picking (**a,** upper panel) and after picking (**a,** bottom panel). The hiPSC colonies were characterized by western blot (**a,** right) to evaluate the expression of ATM protein and the pluripotency marker Oct 3/4 and were compared with primary fibroblasts and MEF feeders. Vinculin was used as a loading control. In **b**, hiPSC colonies were labeled to visualize the expression of the pluripotency markers Oct 3/4, SSEA4 and TRA-1-81. Nuclei were counterstained with DAPI (blue). In **c**, Ctrl and A-T hiPSCs were differentiated *in vitro* into the three germ layers. After 20 days of differentiation, cells were labeled with antibodies specific for *α*-SMA (mesoderm marker), Sox17 (endoderm marker) and *β*-tubulin III (ectoderm marker). Nuclei were counterstained with DAPI (blue). The ability of hiPSCs to generate neuronal cells was confirmed through the formation of floating EBs and rosette formation. Representative images for each differentiation step are shown in **d**

The hiPSC lines were established from primary fibroblasts of two unrelated A-T patients and a healthy control (Ctrl), as detailed in Materials and methods section. Colonies with embryonic stem cell-like morphology were expanded on a mouse embryonic fibroblast (MEF) feeder layer (Figure 1a, left). Western blot analysis revealed, as expected, a positive signal for ATM in fibroblasts and hiPSCs from Ctrl but not from A-T cases (Figure 1a, right). No ATM protein was detectable in MEFs as the antibody used was human specific. Furthermore, only Ctrl and A-T hiPSC lines expressed the pluripotency marker Oct3/4, but not fibroblasts or MEFs (Figure 1a, right). Immunofluorescence analysis attested the pluripotency of the hiPSCs, being positive for Oct3/4, stage-specific embryonic antigen 4 (SSEA4) and Tra-1-81 (Figure 1b). Moreover, the hiPSCs gave rise to the embryonic derivatives ectoderm, endoderm and mesoderm, labeled positive for *β*-tubulin III, Sox17 and alpha-smooth muscle actin (*α*-SMA), respectively (Figure 1c). Also, through embryoid body (EB) and neural rosette formation and subsequent culture in a mitogen-free medium, hiPSCs differentiated into *β*-tubulin III+ and microtubule-associated protein 2+ (MAP2+) neurons (Figure 1d and Supplementary Figure 1).

### Generation of hNPCs and neurons from A-T and Ctrl hiPSCs

**Figure 2:**
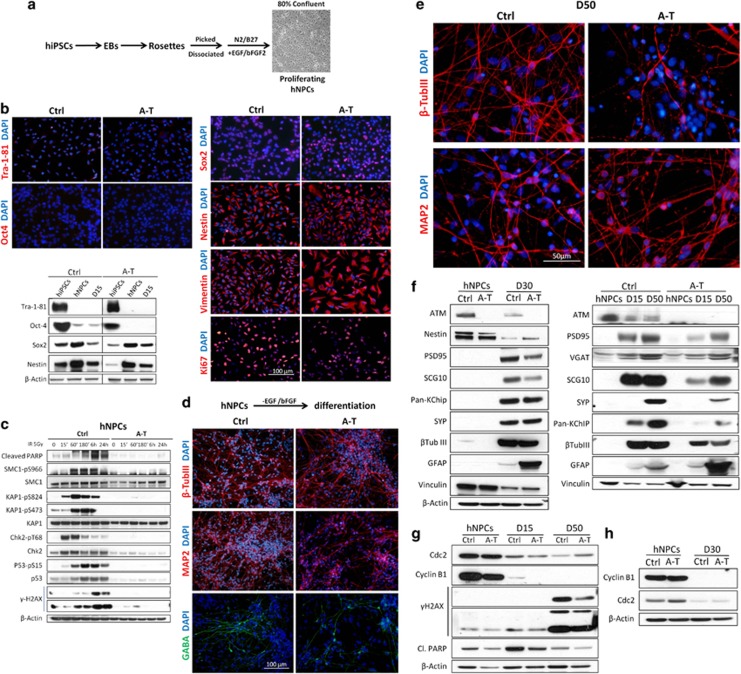
Generation of hNPCs from Ctrl and A-T hiPSCs. To obtain stable and proliferating hNPCs, we followed the protocol depicted in **a** and a representative image of the cell culture obtained is shown. In **b**, the characterization of the hNPCs by immunofluorescence demonstrates the loss of the pluripotency markers Tra-1-81 and Oct3/4, the expression of neural markers (Sox2, Nestin and Vimentin) and the proliferation ability (Ki67). A comparative analysis between hiPSCs and proliferating (hNPCs) and differentiated (D15) hNPCs was performed by western blot. In **c**, proliferating hNPCs were exposed to IR (5 Gy), and the time-dependent DDR activation was evaluated by analyzing the phosphorylation of the indicated ATM substrates, and of cleaved PARP. *β*-Actin was used as a loading control. The real capacity of hNPCs to differentiate into neurons is shown in **d**, where a high number of MAP2+, *β*-tubulin III+ and GABA+ cells are detected at D15 and D50 (**e**). hNPCs and neurons at 15–30 or 50 days of differentiation were collected and analyzed by western blot for the expression of differentiation and maturation markers (**f**) or for the indicated proteins (**g** and **h**)

Proliferating human neural precursor cells (hNPCs) were generated as depicted in Figure 2a. Both Ctrl and A-T hNPCs were negative for the pluripotency markers Tra-1-81 and Oct3/4 (Figure 2b, left) but positive for the neural precursor markers Sox2, Nestin and Vimentin, and for the proliferation marker Ki67 (Figure 2b, right). On western blots, hNPCs showed lower expression of Tra-1-81 and Oct3/4 than hiPSCs, and higher levels of Sox2 and Nestin, which then decreased during neuronal differentiation (Figure 2b, bottom left).

The capacity of hNPCs to activate the DDR was investigated in response to ionizing radiation (IR), which induces DSBs and ATM signalling.^1^ Using phospho-specific antibodies for the indicated substrates of ATM and checkpoint kinase 2 (Chk2; KAP1-pS473), we found that after 15–30 min of IR these molecules were strongly phosphorylated in Ctrl but not in A-T hNPCs (Figure 2c). Interestingly, after IR an accumulation of cleaved poly (ADP ribose) polymerase (PARP), a marker of apoptosis, was detected in Ctrl but not in A-T hNPCs, suggesting that ATM deficiency confers radioresistance in proliferative cells (Figure 2c). When hNPCs were induced to differentiate, they acquired a neuronal-like morphology and at day 15 of differentiation (D15) were positive for *β*-tubulin III or MAP2, with a fraction of cells also expressing the marker of inhibitory neurons *γ*-aminobutyric acid (GABA; Figure 2d), which we were no longer able to detect at D50 in mature neuronal cells^18^ expressing *β*-tubulin III or MAP2 (Figure 2e). Western blot analysis confirmed the downregulation of the stem cell marker Nestin, the upregulation of the neuronal marker *β*-tubulin III at D30 (Figure 2f, left) and at D15 and D50 (Figure 2f, right), and the expression of the postsynaptic marker PSD95 postsynaptic density protein 95, the inhibitory synapse marker vesicular GABA transporter (VGAT), the neuronal growth-associated protein SCG10 and the potassium channel-interacting proteins (KChIPs). At D30 and D50, but not D15, cells also expressed the pre-synaptic marker Synaptophysin (SYP). We detected no obvious differences between Ctrl and A-T hNPCs concerning the generation of neuronal cells, but A-T neurons displayed deficits in the expression of SYP and PSD95 as well as of SCG10 and KChIP, and higher amounts of glial fibrillary acidic protein (GFAP; Figure 2f).

The post-mitotic status of the differentiated cells was confirmed by the downregulation in D15–D50 (Figure 2h) and D30 samples (Figure 2g) of Cdc2 and cyclin B1. Concordant with our previous results,^19^ cleaved poly (ADP ribose) polymerase (PARP) was higher in Ctrl than in A-T neuronal cells. To note, *γ*H2AX (pS139) was also highly expressed at D50, and its levels were lower in A-T than in Ctrl cells (Figure 2g).

### Electrophysiological characterization

**Figure 3:**
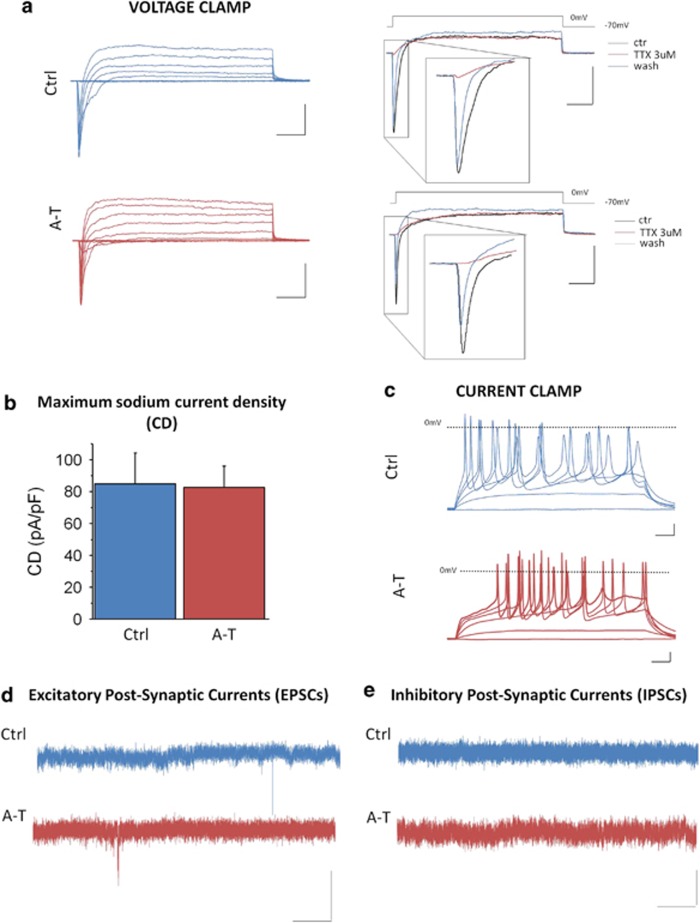
Functional characterization of Ctrl and A-T neurons after 50 days of differentiation. In **a**, left, patch clamp whole-cell recordings of representative total ionic currents elicited with depolarizing voltage steps between –70 and +10 mV (10 mV increments from a holding potential of –70 mV) in Ctrl and A-T hNP-derived neurons; scale bar: 500 pA, 10 ms. In **a**, right, representative currents elicited with a depolarizing voltage step to −10 mV in control (black), during perfusion with TTX 3 *μ*M (red) and after washout (blue) in Ctrl (upper panels) and A-T (lower panels) hNP-derived neurons; scale bar: 500 pA, 10 ms. In **b**, bar graph of maximum sodium current densities (CD) in Ctrl and A-T hNPC-derived neurons (no statistical significant difference: unpaired *T*-test). In **c**, representative action potential discharges recorded in current clamp during injections of 1-s long depolarizing current steps from a holding potential of −70 mV; scale bar: 10 mV, 100 ms. In **d**, traces showing sEPSCs, recorded at the holding potential of −70 mV, while in **e** traces showing the absence of sIPSCs, recorded at the holding potential of +30 mV; scale bar: 10 mV, 1 s

Ctrl and A-T neurons at D53±6 were analyzed by whole-cell patch clamp recordings in order to verify their functional maturation. Voltage clamp experiments showed similar voltage-gated potassium currents (Figure 3a) and, most importantly, voltage-gated sodium currents, selectively blocked by tetrodotoxin (TTX), which is a specific feature of mature neurons (Figure 3a, left panel). Transient peak sodium inward currents had similar amplitude in Ctrl and A-T neurons: maximal peak current density was 84.9±19.6 pA/pF, *n*=10 in Ctrl neurons, 82.7±13.5 pA/pF, *n*=13 in A-T neurons (Figure 3b). Moreover, current clamp experiments showed that both Ctrl and A-T neurons could generate discharges of action potentials in response to injection of depolarizing current steps (Figure 3c). Finally, to determine whether neurons developed functional synapses, we performed voltage clamp experiments for recording spontaneous excitatory and inhibitory postsynaptic currents (sEPSCs and sIPSCs). Both Ctrl and A-T neurons exhibited sEPSCs (Figure 3d) with similar frequencies (0.02±0.01 Hz, *n*=4 cells and 0.03±0.02 Hz, *n*=4 cells, respectively), which were blocked by application of kynurenic acid (3 mM; data not shown), but no sIPSCs (Figure 3e).

### DNA damage-induced G1/S checkpoint in relation to development stage

**Figure 4:**
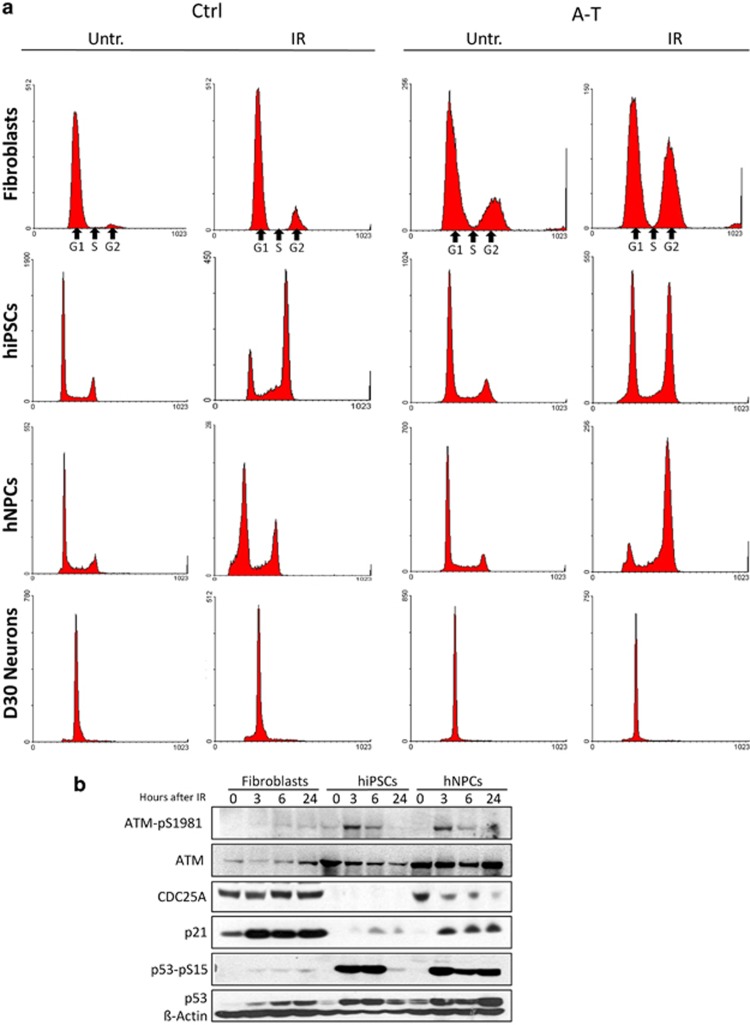
Cell cycle phase distribution changes after IR treatment. Ctrl and A-T cells at various developmental stages (primary fibroblasts, hiPSCs, hNPCs, D30 post-mitotic neurons) were treated with 5 Gy IR, collected after 24 h, and analyzed for DNA content by flow cytometry (**a**). In **b**, Ctrl fibroblasts, hiPSCs and hNPCs were treated with 5 Gy IR, collected at the indicated times and analyzed by western blot. *β*-Actin was used as a loading control

In somatic cells, ATM mediates the G1/S checkpoint but is inactive in ESCs and iPSCs.^20, 21^ To further delineate this activity in relation to the developmental stage, we analyzed the cell cycle profile of primary fibroblast, hiPSCs, hNPCs and terminally differentiated neurons by flow cytometry before and 24 h after IR treatment (Supplementary Figure 2). Primary Ctrl fibroblasts displayed a normal G1/S checkpoint arrest, in contrast to A-T fibroblasts in which this checkpoint defect resulted in a significant increase in the percentage of G2 phase cells (Figure 4a). Notably, not only A-T hiPSCs, but also Ctrl hiPSCs lacked the G1/S checkpoint although, interestingly, Ctrl hNPCs regained it, whereas A-T hNPCs maintained this defect. The analysis of neurons at D30 revealed that all cells were in the G0/G1 phase and this condition was not perturbed 24 h after IR.

To better understand the implication of DDR proteins in the regulation of cell cycle arrest in fibroblasts, hiPSCs and hNPCs, Ctrl cells were harvested at the indicated time points after treatment with 5 Gy IR (Figure 4b) and the expression of ATM-pS1981, p53-pS15, CDC25A and p21^waf1^ was analyzed. Interestingly, although all cell types proficiently activated ATM and induced the accumulation of p53, hiPSCs were unable to induce p21^waf1^, and did not express CDC25A. This could explain the incapacity of hiPSCs to regulate the cell cycle after DNA damage. We also investigated other DDR proteins such as RAD51, the MRN complex (RAD50-MRE11-NBS1) and X-ray repair cross-complementing 1 (XRCC1), and found changes in expression during the developmental stages, suggesting a different ability to repair DNA damage in the different cell types (Supplementary Figure 3).

### Base excision repair capacity in undifferentiated and differentiated hNPCs

**Figure 5:**
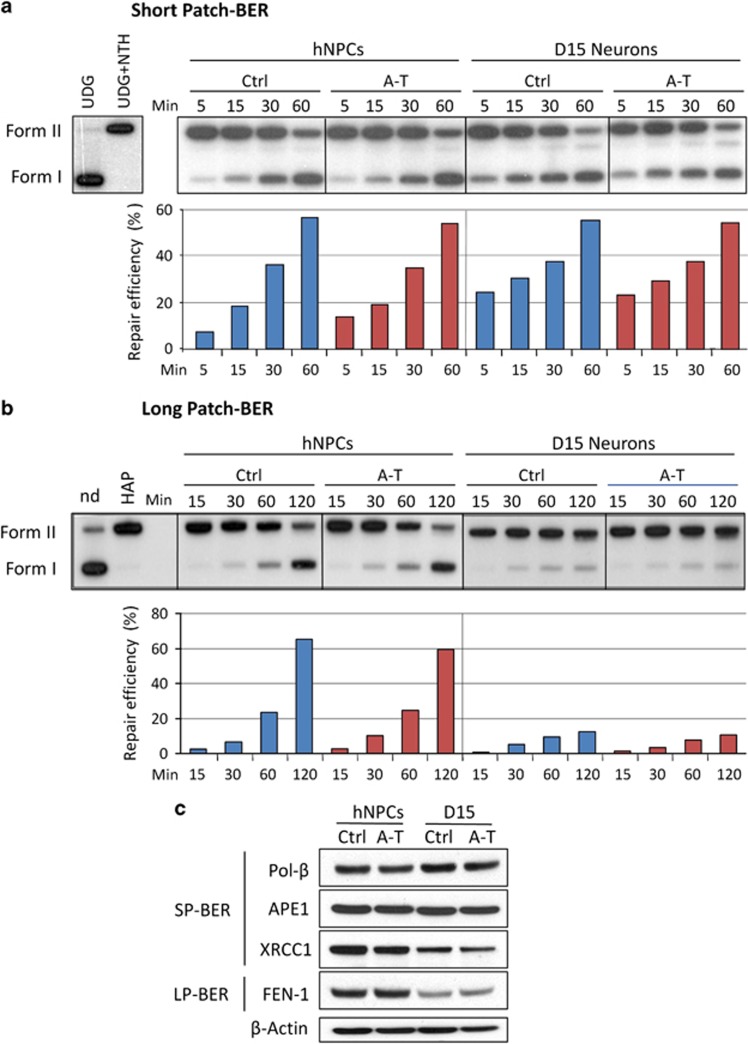
BER activities in proliferating and post-mitotic Ctrl and A-T neuronal cells. *In vitro* repair reactions were performed by using whole-cell extracts and as substrate a 32P-labeled circular plasmid containing a single AP site (pGEM-AP) to measure SP-BER (**a**) or a THF residue (pGEM-THF) for LP-BER (**b**). The correct insertion of a single lesion in the plasmid molecules was checked by digestion with uracil DNA glycosylase (UDG) followed by endonuclease III (NTH) for pGEM-AP (**a**), and by AP endonuclease (HAP1) for pGEM-THF (**b**). Repair kinetics of the AP site (**a**) or THF residue (**b**) by proliferating hNPCs and post-mitotic neurons (D15) were measured following incubation of the single lesion-containing plasmids with extracts from Ctrl or A-T defective neuronal cells for increasing periods of time. Repair products were analyzed by agarose gel electrophoresis and the radioactivity of the bands corresponding to nicked (form II) and supercoiled (form I) plasmids was quantified. Repair efficiency is expressed as relative amount of Form I over total radioactivity in each lane. Blue bar, Ctrl cell extracts; red bar, A-T cell extracts. The expression level of SP-BER and LP-BER proteins was evaluated by western blot in proliferating and post-mitotic neuronal cells (**c**). *β*-Actin was used as a loading control

In neurons, the base excision repair (BER) pathway is essential to remove oxidative DNA damage and single-strand breaks (SSBs) generated by high levels of ROS, and defects in BER contribute to neurodegeneration.^22, 23, 24, 25^ To determine how BER is regulated in pre- and post-mitotic neurons, we analyzed the short-patch (SP) and long-patch (LP) BER subpathways.^23^ Analysis of SP-BER revealed no significant differences between Ctrl and A-T both in the proliferation and post-mitotic (D15) stage (Figure 5a). To better understand this result, we analyzed the levels of the SP-BER proteins apurinic/apyridinic endonuclease 1 (APE1), DNA polymerase *β* (Pol-*β*) and XRCC1, and after differentiation (D15) only the levels of XRCC1 appear reduced (Figure 5c). Similar findings were seen in D30 neurons (data not shown).

LP-BER activity appeared markedly lower in both Ctrl and A-T post-mitotic neurons than in their respective proliferating hNPCs (Figure 5b). This finding is concordant with the reduced expression of Flap endonuclease 1, a protein involved in LP-BER in non-replicating cells (Figure 5c). Overall, these results point out differences that depend on different levels of expression or differential activation of BER enzymes in undifferentiated and differentiated cells, but appear independent of ATM.

### DNA damage response and repair in post-mitotic neurons

**Figure 6:**
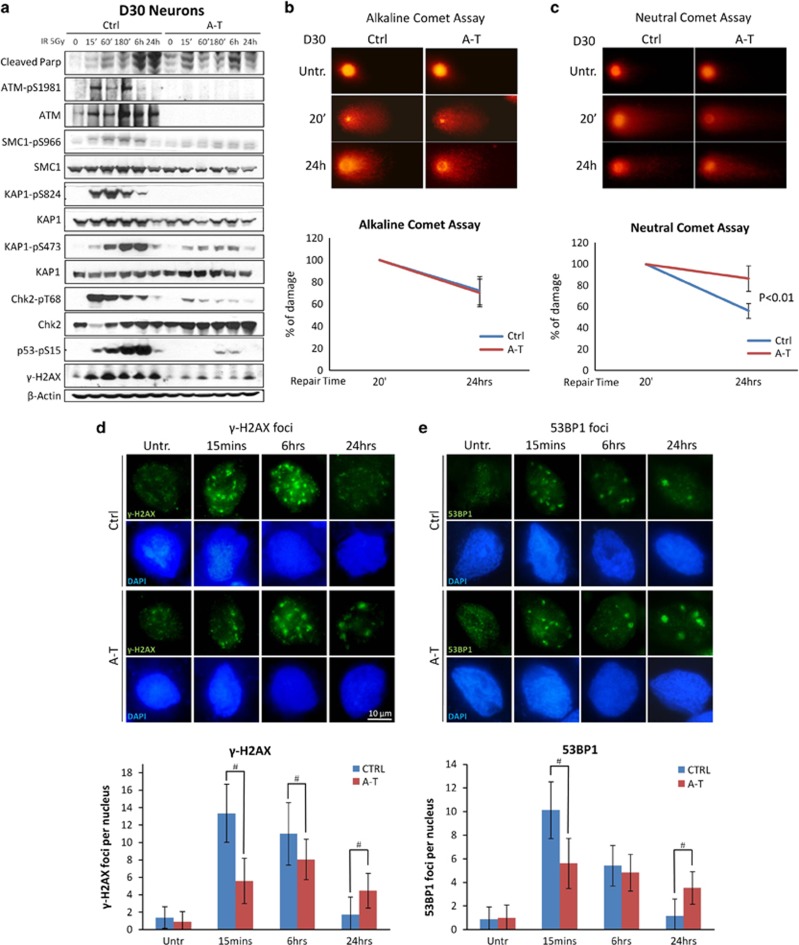
A-T post-mitotic neurons are defective in DDR. In **a**, D30 post-mitotic neurons (D30) were tested by western blot with antibodies specific for the various ATM phosphosubstrates and cleaved PARP at various times after treatment with 5 Gy IR. *β*-Actin was used as a loading control. DNA SSBs (**b**) and DSBs (**c**) were analyzed in neurons (D30) by alkaline and neutral comet assay, respectively, at two different times after treatment. 20 min after H_2_O_2_ or IR treatment was considered the time point with the maximum DNA damage. In **b** and **c**, representative photos of comets from untreated cells and at different time points following treatment. The ratio between treated and untreated tail moments of at least 50–70 cells per experimental point is shown in the graphs (values in %). One representative experiment out of three is shown. In **d** and **e**, formation and resolution of IR-induced nuclear foci. Neurons (D30) were irradiated with 0.5 Gy, collected at the indicated times and labeled for *γ*-H2AX (**d**) and 53BP1 (**e**). For each treatment, the number of foci was scored from 100 cell nuclei per duplicate preparations and from three independent experiments (mean±S.D.) (**d** and **e**, bottom) For each time point, the difference between Ctrl and A-T was statistically significant (^#^*P*<0.01) (analysis performed by the Student's *t*-test)

IR-time course analysis of Ctrl neurons at D30 showed a vigorous ATM-dependent phosphorylation of SMC1-S966, KAP1-S824, Chk2-T68, p53-S15 and *γ*-H2AX, while this response was repressed in A-T neurons (Figure 6a). Similarly, the phosphorylation of the Chk2 substrate KAP1-S473 was abrogated in A-T neurons. Interestingly, as previously described for proliferating hNPCs (Figure 2c), 24 h after IR an accumulation of cleaved PARP was detected in Ctrl but not in A-T neurons, indicating that ATM deficiency confers short-term radioresistance also in terminally differentiated cells (Figure 6a, upper panel). Altogether, these results indicate that normal post-mitotic neurons activate the DDR as efficiently as their proliferating precursors, and that ATM deficiency ablates this response.

The ability of post-mitotic neurons to repair SSBs and DSBs was investigated with the alkaline and neutral comet assay, respectively. Although the repair of SSBs, induced by hydrogen peroxide (H_2_O_2_),^26^ showed no major differences between Ctrl and A-T post-mitotic neurons (Figure 6b), the repair of DSBs, induced by IR,^26^ appeared defective in A-T neurons, which displayed 30% more unrepaired lesions than Ctrl cells (Figure 6c). The latter finding is consistent with a defective DDR, as shown in Figure 6a.

To investigate the role of ATM in the resolution of IR-induced DSBs in D30 neurons, we scored the time-dependent formation and clearance of *γ*-H2AX and 53BP1 nuclear foci by immunofluorescence, as described.^27, 28^ Unirradiated Ctrl D30 neurons showed a basal number of *γ*-H2AX and 53BP1 foci per cell (1.4±1.2 and 0.9±1), which increased to 13.3±3.3 and 10.1±2.4, respectively, after IR, to decline after 6 h and even further after 24 h (Figures 6d and e). Untreated A-T cells showed a number of foci per cell comparable to Ctrl cells, which increased more modestly, 5.6±2.6 for *γ*-H2AX and 5.6±2.1 for 53BP1 after 15 min, and persisted after 24 h (Figures 6d and e graphs). These findings indicate that A-T post-mitotic neurons show slower kinetics of DSBs repair than Ctrl, concordant with previous findings in other ATM-deficient cells.^19^

The apoptotic effect of radiation on Ctrl and A-T neurons 24 h after 5 Gy IR was assessed by flow cytometry analysis of the subdiploid DNA peak (Supplementary Figure 4). Fewer apoptotic cells were detected in IR-treated A-T cells (22.2%) than in Ctrl cells (32.3%), suggesting that ATM deficiency attenuates short-term apoptosis, concordant with the findings on cleaved PARP (Figure 6a) and our previous findings.^19, 29^

### Treatment of A-T and Ctrl neuronal cells with genotoxic and oxidative agents

**Figure 7:**
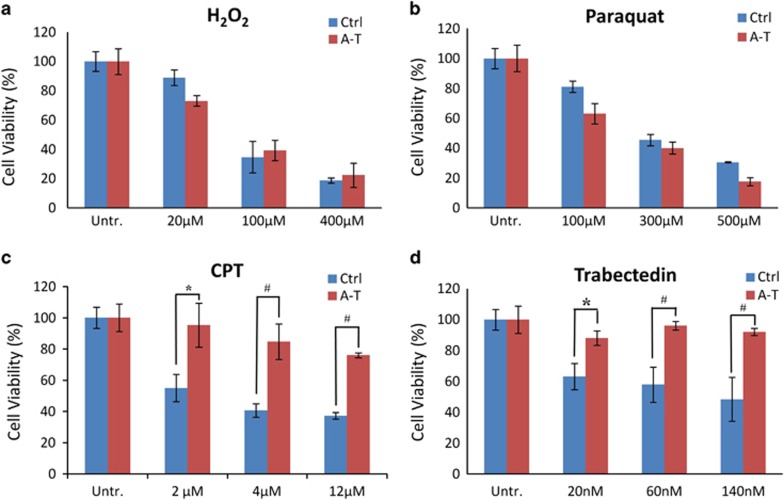
A-T post-mitotic cells are resistant to CPT and trabectedin treatment. Neurons at D30 grown in 96-well plates were assessed for cell viability by CellTiter-Glo 72 h after exposure to H_2_O_2_ for 20 min (**a**), or continuously with Paraquat (**b**), CPT (**c**) and trabectedin (**d**) at the indicated doses. All treatments were performed in triplicate wells. The graphs show mean±S.D. and, where indicated, the difference between Ctrl and A-T was statistically significant (*P<0.05; ^#^*P*<0.01) (analysis performed by the Student's *t*-test)

As genotoxic agents and oxidative stress may similarly affect ATM-deficient neurons, we investigated the viability of Ctrl and A-T post-mitotic neurons (D30) upon treatment with compounds that work through different mechanisms: camptothecin (CPT), which inhibits Topoisomerase I (Top1) and traps Top1-DNA covalent complexes (Top1-ccs);^30^ trabectedin, which blocks transcription factor activity;^31^ Paraquat, which induces ROS through depolarization of the inner mitochondrial membrane,^32^ and H_2_O_2_. Ctrl and A-T post-mitotic neurons were equally sensitive to H_2_O_2_ and Paraquat (Figures 7a and b), a finding concordant with previous data^33^ and with the results in Figure 6b showing that these cells have the same ability to resolve SSBs. By contrast, A-T neurons were significantly more resistant than Ctrl neurons to CPT and trabectedin by about 40% and 30%, respectively (Figures 7c and d).

### Accumulation of Top1-ccs in A-T post-mitotic neurons

**Figure 8:**
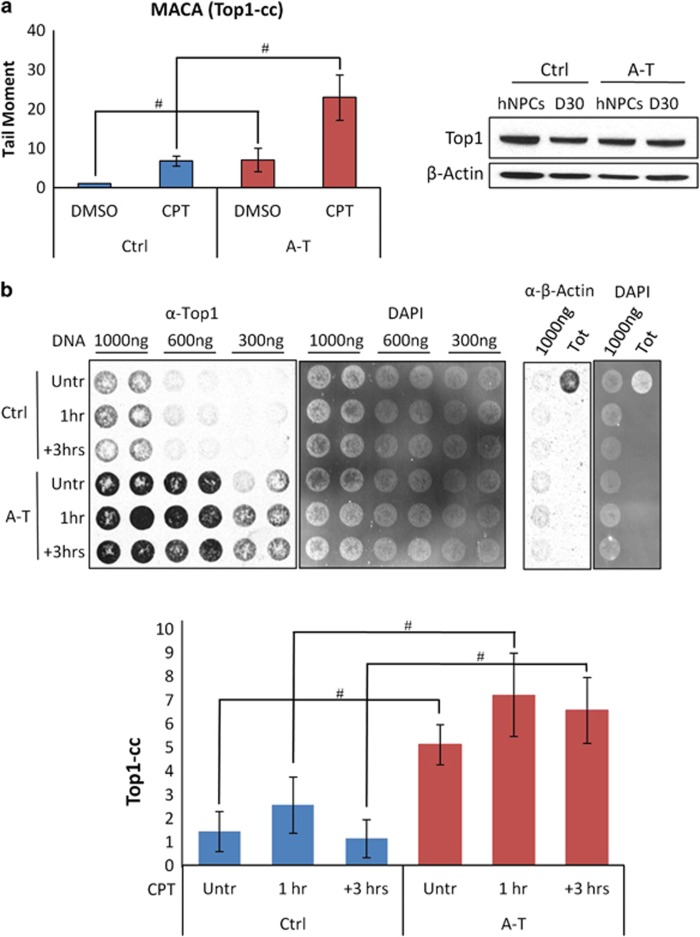
A-T post-mitotic neurons accumulate abnormal levels of Top1-cc. Total intracellular levels of Top1 protein were analyzed by western blot in proliferating hNPCs and post-mitotic neurons (**a** left). The levels of endogenous Top1-ccs were quantified in Ctrl and A-T post-mitotic neurons (D30) subjected to 30 *μ*M CPT for 40 min by MACA from 50 cells per sample per experiment (**a** right). Data are the average of three independent experiments (mean±S.D.). For each time point, the difference between Ctrl and A-T was statistically significant (^#^*P*<0.01) except where indicated. In **b**, Ctrl or A-T post-mitotic neuronal cells were untreated or treated with 30 *μ*M CPT for 60 min or incubated and harvested 3 h after washing. The DNA was purified from cells after lyses with a denaturing buffer (MB) as reported in Materials and methods section. The indicated amount of DNA was vacuum blotted on a nitrocellulose membrane, which was then tested with anti-Top1 antibody and ECL (left) and with DAPI (middle). *β*-Actin (left) represents the control for non-covalently bound contaminant proteins. The densitometry analysis of Top1-cc signals after normalization for blotted DNA (DAPI) from three biological replicates (mean±S.D.) is reported in the graph. Y axis represents the relative signal intensity. Where indicated the difference between Ctrl and A-T was statistically significant (^#^*P*<0.01; analysis performed by the Student's *t*-test)

As in murine neural cells ATM deficiency results in accumulation of Top1-ccs and failure to recover global transcription after Top1-cc trapping,^34^ we used a modified alkaline comet assay (MACA),^34^ which indirectly quantifies Top1-ccs, to analyze hNPC-derived neurons (D30) untreated or treated for 45 min with 30 *μ*M CPT (Figure 8a, left). As shown in the graph, the accumulation of Top1-ccs was much greater in A-T than in Ctrl neurons, both before and after CPT treatment. Of note, the overall levels of Top1 in proliferating and post-mitotic neurons (D30) were similar (Figure 8a, right).

To further confirm these findings, we applied the rapid approach to DNA adduct recovery (RADAR) assay that allows to directly detect Top1-ccs bound to genomic DNA.^35^ D30 neurons, incubated for 1 h with 30 *μ*M CPT, were harvested immediately or 3 h after washout of the drug to analyze the recovery of the damage. DNA was isolated, dot blotted and Top1-ccs were revealed with an anti-Top1 antibody and ECL. The levels of Top1-ccs (Figure 8b, left) were then normalized with the total amount of DNA, revealed by 4′,6-diamidin-2-fenilindolo (DAPI; Figure 8b, middle). *β*-Actin was used to verify that the DNA samples were free of non-covalently bound contaminating proteins (Figure 8b, right). The graph shows Top1 protein levels after normalization with DNA, and, as expected, untreated A-T neurons displayed higher accumulation of Top1-ccs than Ctrl cells, and also appeared defective in Top1-cc recovery after CPT removal.

## Discussion

The mechanisms underlying neurodegeneration in A-T still remain elusive. Up to the present, ATM knockout and knockin mice^36, 37^ and *in vitro* ATM-deficient human neural stem cell (hNSC) models^19, 38^ have been useful for elucidating many aspects of the neuropathology, but animal models do not recapitulate the CNS disease,^39^ while hNSCs have been argued to be very heterogeneous.^40^ To obtain a more reliable *in vitro* model of neurodegeneration in A-T, two novel approaches have recently been described: the establishment of patient olfactory mucosa-derived neurospheres, which give rise to neurons,^41^ and the reprogramming of patient fibroblasts to a pluripotent stage.^15, 17^

As a functional analysis of the neuronal cells harboring patient-specific mutations in ATM has not yet been reported, in this study we examined hiPSC-derived A-T hNPCs and terminally differentiated neurons in culture. We found that A-T hNPCs displayed a strongly attenuated response to DSBs with respect to Ctrl hNPCs, which is concordant with our previous study on hNSCs.^19^

Moreover, terminally differentiated A-T neurons exhibited decreased expression of SYP and PSD95, which is concordant with previous works showing pre- and postsynaptic degeneration in ATM knockout mice,^42^ the requirement of cytoplasmic ATM for phosphorylation of the synaptic vesicle proteins VAMP2 and Synapsin-1, and that ATM deficiency affects spontaneous vesicle release and establishment of long-term potentiation.^8^ We also showed that A-T neurons are defective in the expression of SCG10 and KChip, altogether underpinning defects in neuronal maturation.

In agreement with the role of ATM in establishing the G1/S checkpoint arrest to prevent cells with damaged DNA from entering the S-phase,^1^ primary Ctrl fibroblasts effectively arrested at the G1/S boundary after IR, but once reprogrammed into hiPSCs lost this G1/S checkpoint arrest to regain it at the stage of hNPCs. Instead, A-T cells showed a disrupted G1/S checkpoint, whatever the developmental stage. Notably, the absence of the G1/S checkpoint arrest in Ctrl hiPSCs is a characteristic of ESCs, where the cells subjected to DNA damage in G1 enter S-phase to be eliminated by apoptosis, thus avoiding the propagation of mutations detrimental for the whole organism.^43, 44^ Accordingly, hiPSCs showed a great accumulation of p53-pS15 and faint induction of p21^waf1^, compatible with a p53-dependent apoptotic response rather than with a G1/S checkpoint activation. Moreover, we found that Ctrl hiPSCs upregulate homologous recombination components such as Rad51, concordant with the fact that ESCs predominantly use this high fidelity pathway to avoid the accumulation of mutations, in contrast to somatic cells, which rely on the error-prone non-homologous end joining to repair DNA damage occurring in G1.^45^

BER is the major pathway for repair of oxidatively damaged DNA and various SSB intermediates,^22^ and previous findings demonstrate that ATM and Chk2 facilitate the recruitment of downstream BER proteins to the initial damage recognition/excision step to promote BER.^46^ Here, we found no significant differences in SP-BER activity between Ctrl and A-T proliferative hNPCs and post-mitotic neurons, while conversely, LP-BER activity was reduced in post-mitotic neurons. It should be noted that both SP-BER and LP-BER enzymatic efficiencies have been found to decrease, along with the respective protein components, in a neuroblastoma cell line upon differentiation,^24^ and while our data on LP-BER concord with these results, those on SP-BER are clearly at variance. This could be because the expression of the SP-BER component XRCC1 is totally ablated in differentiated neuroblastoma cells,^24^ while being only partially downregulated in our neurons. Regardless of this discrepancy, the variations in LP-BER activity between post-mitotic neurons and proliferative hNPCs were totally independent of ATM expression.

In accordance with our previous findings using hNSCs,^19^ here we found that the DDR was vigorously activated in post-mitotic Ctrl neurons, according to the strong phosphorylation of ATM substrates, whereas in A-T post-mitotic neurons this response was almost ablated. We also highlighted relevant DSB repair defects in A-T neurons, which indicated that ATM deficiency delays the repair of DSBs, in accordance with previous reports^19, 47^ and with the observation that ATM is required for the repair of heterochromatic DSBs.^48^ Furthermore, we demonstrated that A-T neurons show less apoptosis-related sub-diploid DNA content and a lower cleaved PARP induction after IR-treatment than Ctrl neurons. Indeed, ATM deficiency has been found to attenuate the apoptotic response to IR in A-T lymphoblastoid cells and in hNSCs^19, 29^ and ATM is required for the p53-mediated apoptosis of developing post-mitotic neurons exposed to IR.^49, 50^

Although ATM appears involved in sensing and modulating the cellular response to ROS,^7^ in our study A-T post-mitotic neurons were as sensitive to oxidative agents as Ctrl post-mitotic neurons, in agreement with the alkaline comet assays showing that these neurons equally repair SSBs, and in the case of H_2_O_2_, with results obtained with A-T lymphoblastoid cells.^33^ By contrast, A-T post-mitotic neurons were significantly more resistant than Ctrl neurons to the genotoxic agents CPT and Trabectedin, probably because, in the absence of ATM, drug-induced DSBs in transcribed genes fail to activate a p53-dependent apoptotic response. This finding is concordant with results showing that ATM inhibition suppresses the genotoxic response in rat cerebellar granule neurons.^51^

Finally, as it has been recently shown that post-mitotic neural cells from ATM-deficient mice accumulate Top1-ccs and fail to recover global transcription after Top1-cc trapping by CPT,^34^ and given that Top1-ccs can promote transcription elongation arrest and decay,^52^ a phenomenon underlying neurodegeneration,^53, 54, 55^ we analyzed Top1-ccs by two different assays and found greater levels of these intermediates in A-T than in Ctrl neurons. This finding, which to our knowledge has never been reported in human A-T neurons, warrants further studies to determine the impact of Top1-cc on genome-wide global transcription.

In conclusion, we have shown that A-T post-mitotic neurons display normal electrophysiological activity, defective expression of maturation markers, attenuated response to and repair of DSBs, but normal capacity to repair SSBs and normal BER activities. Strikingly, A-T neurons exhibit elevated levels of Top1-ccs, which may potentially impair transcriptional fidelity, a possibility that requires further investigation. Finally, we have shown that hiPSCs, like ESCs, provide a unique *in vitro* model to study the G1/S cell cycle checkpoint in a developmental context.

## Materials and Methods

### Primary cell culture

A-T patient-derived primary dermal fibroblasts (GM03487 and GM02530) were purchased from the Coriell Institute for Medical Research (Camden, NJ, USA). Both carry compound heterozygous mutations in *ATM* gene: GM03487 (c.8266A>T and c.1141_1142ins4), GM02530 (c.5675_5762del in trans with c.2251-1G>A and c.6573_6653del). Dermal fibroblasts of a healthy, ethnically matched donor and their iPSCs derivatives were previously described by us.^18^ Fibroblasts were cultured at 37 °C in a 5% CO_2_ in E-MEM supplemented with 15% heat-inactivated FCS, 100 units/ml penicillin, 100 *μ*g/ml streptomycin, 100 *μ*M non-essential amino acids and 2 mM glutamine. Primary MEFs (PMEF-CFL, Millipore, Bedford, MA, USA) were cultured in DMEM/F12 supplemented with 20% heat-inactivated FCS, 100 units/ml penicillin, 100 *μ*g/ml streptomycin, 100 *μ*M non-essential amino acids and 2 mM glutamine. PMEF-CFLs were mitotically inactivated (Mito-MEFs) with 1 *μ*g/ml Mytomycin C (Kiowa Hakko Kirin, Milan, Italy) in DMEM high glucose (Euroclone, Pero, Italy) for 3 h and were then collected and plated into 0.1% gelatin-coated six-well plates at a concentration of 1.8 × 10^5^ cells/cm^2^.

### hiPSC generation and culture

Human primary fibroblasts were infected with the STEMCCA Cre-Excisable Constitutive Polycistronic Lentivirus (Millipore) following the manufacturer's instructions. After 20–25 days, hiPSC clones were picked and transferred onto a Mito-MEF feeder in human embryonic stem cell (hESC) medium composed of DMEM/F12 supplemented with 20% KnockOut serum replacement (Life Technologies, Monza, Italy), 100 units/ml penicillin, 100 *μ*g/ml streptomycin, 100 *μ*M non-essential amino acids, 2 mM glutamine, 1 mM sodium pyruvate, 100 *μ*M 2-mercaptoethanol and 20 ng/ml basic fibroblast growth factor (bFGF; Peprotech, Rocky Hill, NJ, USA). For passaging, hiPSCs were incubated with DMEM/F12 containing 1 mg/ml collagenase IV (Sigma, Milan, Italy) at 37 °C for 10 min. Cells were then scraped, collected and transferred at a split ratio of 1 : 3 to a new plate on Mito-MEFs in hESC medium. For feeder-free culture, hiPSCs were seeded on Matrigel (hESC-qualified matrix, BD Biosciences, San Jose, CA, USA)-coated plates in Nutristem culture medium (Stemgent, Cambridge, MA, USA) supplemented with 100 units/ml penicillin and 100 *μ*g/ml streptomycin. For differentiation into derivatives of all three primary germ layers, hiPSCs were collected as for passaging and transferred to a gelatin-coated plate in DMEM/F12 supplemented with 20% heat-inactivated FCS, 100 units/ml penicillin, 100 *μ*g/ml streptomycin, 100 *μ*M non-essential amino acids and 2 mmol/l glutamine.

### Generation of hNPCs and terminal differentiation

hiPSC colonies were harvested as for passaging, resuspended in hESC medium without bFGF and plated into a 6 cm low binding dish (HydroCell, Nunc, Cornaredo, Italy) for floating cultivation. After 5 days in suspension culture, EBs were collected and plated into a Matrigel (growth factor reduced, BD Biosciences)-coated dish for additional 7 days in hESC media supplemented with 1X N2 (Gibco, Monza, Italy). After 7 days, rosettes were manually picked, mechanically dissociated into single cells and resuspended in Neural Precursor Medium (NPMedium) composed of DMEM/F12 supplemented with 2 mM glutamax, 100 units/ml penicillin, 100 *μ*g/ml streptomycin, 1 : 500 B27 (Gibco), 1X N_2_, 20 ng/ml epidermal growth factor (EGF) and 20 ng/ml bFGF (Società Italiana Chimici, Roma, Italy) and plated into Matrigel-coated flasks. NPMedium was changed every 2 days. For passaging, 90% confluent cells were detached using Accutase (PAA; Piscataway, NJ, USA) and split at a ratio of 1 : 4. hNPCs displayed a homogenous population starting at passage 3. To obtain terminally differentiated neurons, proliferating hNPCs were plated in NPMedium at a concentration of 5 × 10^3^ cells/cm^2^; 24 h later medium was changed to NPMedium without bFGF and EGF and replaced every 2–3 days thereafter.

### Western blotting

Western blot analysis was performed as previously described.^56^ Briefly, cells were lysed in Laemmli buffer (0.125 M Tris-HCl pH 6.8, 5% SDS) and lysates were sonicated, size-fractionated by SDS-PAGE and electroblotted onto PVDF membranes (Millipore), which were then incubated with primary antibodies and binding detected by ECL (Pierce, Rockford, IL, USA) on autoradiographic films. Bands were acquired with a digital scanner. The primary mouse monoclonal antibodies used were against the following molecules: OCT3/4(clone C-10), p53, Cdc2 (p34), p21^waf1^ (Santa Cruz Biotechnology, Inc., Dallas, TX, USA), *β*-tubulin III (clone 2G10), SYP, Vinculin, *β*-actin (Sigma), Tra-1-81 (eBioscience, San Diego, CA, USA), anti-Sox2, CDC25A (Abcam, Cambridge, UK), cyclin B1 (clone GNS-1, BD Pharmigen, Franklin Lakes, NJ, USA), *γ*H2AX (H2AX-pSer139, clone JBW301, Millipore), PSD95, SCG10, Pan-KChIP, SYP (NeuroMab, Davis, CA, USA), Nestin, MAP2 (clone AP20, Chemicon International, Billerica, MA, USA), KAP1 (TIF1*β*, clone 4E1, Cell Signaling Technology, Danvers, MA, USA), Chk2 (clone DCS270-273, MBL Intl Corp., Des Plaines, IL, USA) and Top1 (BD Biosciences); rabbit antibodies specific for ATM (clone Y170, Epitomics, Burlingame, CA, USA), ATM pS1981 (Rockland Inc., Gilbertsville, PA, USA), cleaved PARP, XRCC1, KAP1-pS824, Chk2-pThr68, p53-pSer15 (Cell Signaling Technology), GFAP (Millipore), VGAT (Synaptic System, Goettingen, Germany), Pol*β*, Fen1 (Abcam), APE1 (Santa Cruz Biotechnology, Inc.), SMC1, SMC1-pSer966 (Bethyl Laboratories, Inc., Montgomery, TX, USA) and KAP1-pS473 (Biolabs, San Diego, CA, USA).

### Immunofluorescence

Cells grown on coverslips were fixed in 4% buffered paraformaldehyde and permeabilized with 0.1% Triton X-100 in PBS for 10 min.^19^ After blocking with PBS plus 10% normal goat serum for 40 min, cells were incubated overnight with primary antibodies at 4 °C. The following primary antibodies were used: monoclonal anti-Oct 3/4 (Clone C-10, Santa Cruz Biotechnology, Inc.), anti-SSEA4 (eBioscience), anti-Tra-1-81 (eBioscience), anti-*α*-SMA (clone 1A4, Sigma), anti-Sox17 (clone 245013, R&D System, Minneapolis, MN, USA), anti-*β*-tubulin III (clone TUJ1, Covance, Princeton, NJ, USA), anti-Nestin, anti-MAP2 (clone AP20, Chemicon International), anti-Vimentin (DAKO, Cernusco sul Naviglio, Italy), and polyclonal anti-GFAP (Millipore), anti-GABA, and anti-Ki67 (Thermo Scientific, Milan, Italy). After three washes with PBS, cells were incubated with Alexa fluor-labeled goat anti-mouse or anti-rabbit antibodies (Life Technologies) for 45 min. Cells were washed, counterstained with DAPI, mounted on glass-slides with Prolong Gold Antifade (Life Technologies) and analyzed using an Eclipse E1000 Nikon fluorescence microscope equipped with a DMX1200F CCD camera (Torino, Italy). For the assessment of nuclear foci, cells on coverslips were washed with 0.9% NaCl, air dried, fixed in 3% buffered paraformaldehyde and permeabilized for 5 min at 4 °C in 0.5% Triton. Cells were then blocked in PBS containing 5% BSA and 0.2% Tween20, labeled with antibodies specific for H2AX-pS139 antibody (clone JBW301, Upstate Biotechnology, New York, NY, USA) and 53BP1 (NB100-304; Novus Biologicals, Cambridge, UK) and foci from three independent experiments and duplicate slides were enumerated.

### Electrophysiology analysis

Electrophysiological recordings were done as reported^18, 57^ at room temperature (22–25 °C) using a Multiclamp 700 A patch clamp amplifier and pClamp 10.2 software (Molecular Devices, Sunnyvale, CA, USA). Recordings usually started 5 min after the rupture of the membrane patch, to allow intracellular dialysis with the pipette solution. External bath solution consisted of 129 mM NaCl, 1.25 mM NaH_2_PO_4_, 35 mM glucose, 1.8 mM MgSO_2_, 1.6 mM CaCl_2_, 3 mM KCl and 10 mM HEPES, pH 7.4 with NaOH. The internal pipette solution consisted of 120 mM K gluconate, 15 mM KCl, 2 mM MgCl_2_, 0.2 mM EGTA, 10 mM HEPES, 20 mM P-creatine, 2 mM ATP-Na_2_, 0.2 mM GTP-Na_2_ and 0.1 mM Leupeptine, pH 7.2 with KOH. Cell capacitance and series resistance errors were carefully compensated (∼85%) throughout the experiment. The remaining linear capacity and leakage currents were eliminated online using a P/4 subtraction paradigm. Pipette resistance was between 2.6 and 3.0 MΩ. For the recordings of total voltage-gated ion currents, signals were filtered at 10 kHz and sampled at 100 kHz. For the recordings of postsynaptic currents, signals were filtered at 3 kHz and sampled at 10 kHz. When we switched the amplifier to current clamp mode, we applied the bridge balance compensation and held the resting potential at −70 mV by injecting the appropriate holding current. Neuronal firing was recorded injecting depolarizing current pulses of increasing amplitude. The neurons with unstable resting potential and/or unstable firing were discarded. In current clamp mode signals were filtered at 10 kHz and sampled at 20 kHz.

### Cell cycle phase analysis

Cells at different developmental stages were irradiated with 5 Gy IR, incubated for 24 h and then fixed with 70% ethanol, washed, treated PBS-RNAse A, at 37 °C for 30 min and finally stained with propidium iodide. Cells were analyzed with a FACSCalibur flow cytofluorimeter instrument fitted with a Cell Quest software package (Becton Dickinson, Sunnyvale, CA, USA).

### *In vitro* BER assay

Whole-cell extracts for BER assays were prepared from 15 × 10^6^ cells that were harvested, resuspended in 400 *μ*l buffer I (10 mM Tris pH 7.8, 200 mM KCl) and then lysed by adding an equal volume of Buffer II (10 mM Tris pH 7.8, 200 mM KCl, 2 mM EDTA, 40% glycerol, 0.2% Nonidet-P40, 2 mM DTT and protease inhibitors). After stirring for 1 h at 4 °C and centrifugation, the supernatant was aliquoted and stored at −80 °C. Closed circular DNA molecules containing a single lesion were constructed as described^58^ by priming single-stranded pGem-3Zf(+) DNA with the oligonucleotides containing the modified base of interest (uracil and tetrahydrofuran (THF). These oligonucleotides were [*γ*-32P]ATP 5′ end-labeled. The *in vitro* DNA synthesis was performed by using DNA T4 DNA polymerase holoenzyme, single-stranded DNA-binding protein, dNTPs and T4 DNA ligase. The plasmid DNA containing a single uracil residue was digested with UDG to produce a single abasic site. Repair of the plasmid DNA containing the lesions (pGEM-AP/THF) was conducted as described.^58^ Briefly, reaction mixtures (50 *μ*l) contained 40 mM HEPES/KOH (pH 7.9), 75 mM KCl, 5 mM MgCl_2_, 0.5 mM dithiothreitol, 50 *μ*M of each dNTP, 2 mM ATP, 40 mM phosphocreatine, 2.5 *μ*g of creatine phosphokinase (type I), 3.4% glycerol, 18 *μ*g of bovine serum albumin and 50 *μ*g of cell extracts. After incubation at 30 °C, the plasmid DNA was recovered and loaded onto agarose gels. The relative yield of the different plasmid forms was measured. All experiments were repeated at least three times, and representative experiments are shown.

### Comet assay and MACA

To prepare the Comet slides, the day before analysis microscope glass slides were washed with methanol, air dried and immersed in molten Normal Melting-Agarose (1%) to obtain the first agarose layer, before being stored overnight at 4 °C. To evaluate DNA strand breaks, post-mitotic neurons were incubated with 20 *μ*M H_2_O_2_ (Sigma) for 20 min or irradiated with 5 Gy. After treatment with Accutase, the cell suspension was added to 180 *μ*l of molten Low Melting-Agarose (0.7% LM-agarose) at 37 °C, and immediately layered onto Comet slides, covered with a coverslip and stored at 4 °C in the dark. After 25 min, the coverslip was removed and a second LM-Agarose layer was layered onto the slides. The slides were then transferred to a pre-chilled lysis solution (2.5 M NaCl, 100 mM EDTA, 10 mM Tris-base, 300 mM NaOH, pH 10, and 1% freshly added Triton X-100) for 2 h at 4 °C in the dark. In the alkaline condition, the slides were placed in a horizontal electrophoresis chamber and the denaturation step was performed in pre-chilled electrophoresis solution (300 mM NaOH, 1 mM EDTA, pH>13) at 4 °C in the dark. After 20 min, the slides were electrophoresed (1 V/cm, 300 mA) for 20 min, and washed with neutralization buffer (0.4 M Tris-HCl, pH 7.4). In the neutral condition, after the lysis the slides were washed twice with 1 × Tris-borate EDTA buffer solution pH 8.3 (TBE) for 10 min. Electrophoresis was carried out at the rate of 1.0 V/cm for 20 min. The slides were then washed in deionized water for 5 min. MACA on post-mitotic neurons was performed as described.^34^ After electrophoresis, Comet slides were fixed with pre-chilled methanol, air dried overnight, stained with ethidium bromide (0.1 mg/ml) for 10 min, and images acquired with an Eclipse E1000 Nikon fluorescence microscope equipped with a DMX1200F CCD camera. Comet images were processed with Comet Score software (v1.5; TriTek Corporation, Sumerduck, VA, USA).

### Rapid approach to DNA adduct recovery

Post-mitotic neurons (D30) were incubated with or without 30 *μ*M CPT for 1 h. Following treatment, part of the cells was immediately lysed, while the remaining washed with PBS and incubated in fresh medium for 3 h before lysis. For DNA–protein covalent complexes isolation, cells were lysed in the culture plates by addition of 2 ml of MB lysis reagent (6 M GTC, 10 mM Tris-HCl, pH 6.5, 20 mM EDTA, 4% Triton X100, 1% Sarkosyl and 1% dithiothreitol) and processed as described.^35^ Purified DNA was vacuum blotted on nitrocellulose membranes and Top1 detected using a mouse monoclonal anti-Top1 antibody (BD Biosciences) and ECL. Sample loadings were normalized for DNA content using DAPI.

### Neuronal viability assay

Neuronal viability was evaluated using the Cell Titer-Glo luminescent assay (Promega, Madison, WI, USA). Briefly, hNPCs were seeded into Matrigel-coated 96-well plates (10 000 cells per well) and let to differentiate for 30 days, after which they were treated in triplicate with CPT, H_2_O_2,_ Paraquat (all from Sigma) or trabectedin (Yondelis; ET-743) for 72 h. H_2_O_2_ was removed after 20 min. Afterward, a volume of CellTiter-Glo Reagent was added to each well and luminescence measured using a Tecan Genios instrument (Männedorf, Switzerland).

## Abbreviations

ATM: ataxia telangiectasia mutated
Chk2: checkpoint kinase 2
SSEA4: stage-specific embryonic antigen 4
*α*-SMA: *α*-smooth muscle actin
MAP2: microtubule-associated protein 2
PARP: poly (ADP ribose) polymerase
GABA: *γ*-aminobutyric acid
PSD95: discs large homolog 4
VGAT: vesicular GABA transporter
KChIP: Kv channel-interacting protein 1
GFAP: glial fibrillary acidic protein
XRCC1: X-ray repair cross-complementing 1
CPT: camptothecin
DAPI: 4′,6-diamidin-2-fenilindolo
APE1: apurinic/apyridinic endonuclease 1
Pol-*β*: DNA polymerase *β*
bFGF: basic fibroblast growth factor
EGF: epidermal growth factor
NPCs: neural precursor cells
hiPSCs: human-induced pluripotent stem cells
DDR: DNA damage response
DSBs: double-strand breaks
Top1-cc: topoisomerase 1-DNA covalent complexes
IR: ionizing radiation
