# Supplementary material for: Functional and molecular defects of hiPSC-derived neurons from patients with ATM deficiency
Source: Cell Death Dis. 2014 Jul 17;5(7):e1342–. doi: 10.1038/cddis.2014.310 (PMC4123100; doi:10.1038/cddis.2014.310)
Supplement: Supplementary Information [file cddis2014310x1.pdf]

Carlessi et al, Functional and molecular defects of hiPSC-derived neurons from patients with ATM deficiency

Supplementary Fig. 1

hiPSCs were able to generate differentiated neurons through embryoid body and neural rosette formation and subsequent culture in a mitogen-free medium containing N2. After 10 days, a largenumber of β-Tubulin III and Map2 positive cells were present whereas only few GFAP-expressingcells were detected.

Supplementary Fig. 2

The G1/G2-M ratio before and 24 h after IR treatment is shown for each cell type and determined from the flow cytometric analysis of the DNA histograms depicted in Fig 4.

Supplementary Fig. 3

Primary fibroblasts, hiPSCs, proliferating hNPCs and postmitotic neurons (D30) were compared byWestern blot for the expression of the DDR proteins RAD51, RAD50, MRE11, NBS1, and XRCC1. β-Actin was used as a loading control.

Supplementary Fig. 4

D30 post-mitotic Ctrl and A-T cells were treated with 5 Gy IR, collected after 24 h and analyzed for DNA content by flow cytometry. The percentage of sub-diploid apoptotic cells is indicated.

Sup. Fig.1

Representative images of hiPSCs directly differentiated into neuronal cells

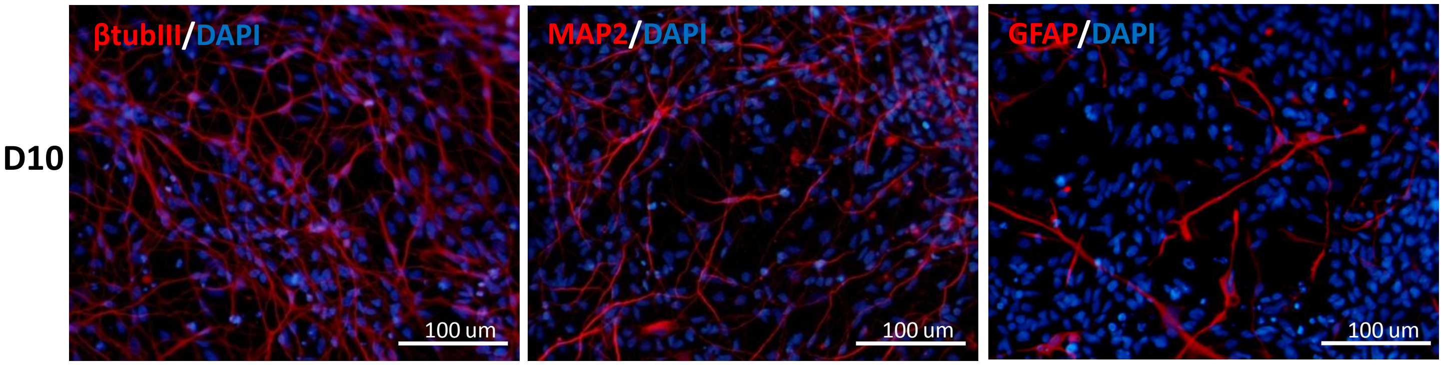

Sup. Fig.2

	Ctrl		A-T	
	G1/G2-M Untreated	G1/G2-M (5Gy, 24 h)	G1/G2-M Untreated	G1/G2-M (5Gy, 24 h)
Fibroblast	16.4	7	3.7	1.5
hiPSCs	2.7	0.3	3.2	0.76
hNPCs	4.1	1.7	3.4	0.07
Post-Mitotic (D30)	29.1	23.64	14.4	16.2

Sup. Fig.3

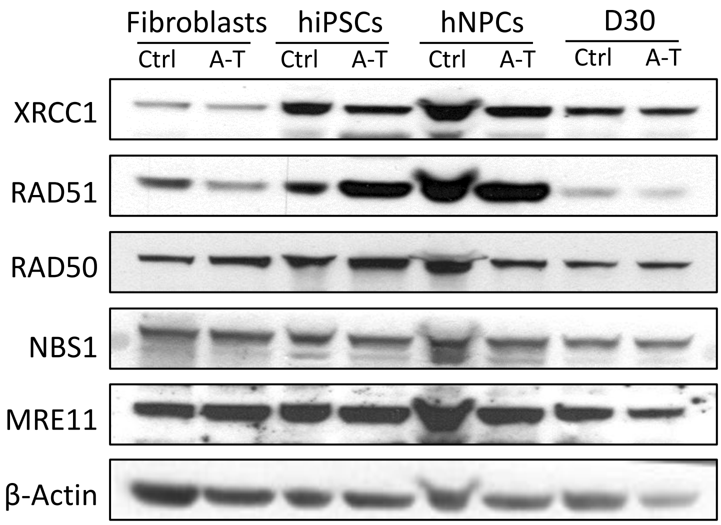

Sup. Fig.4

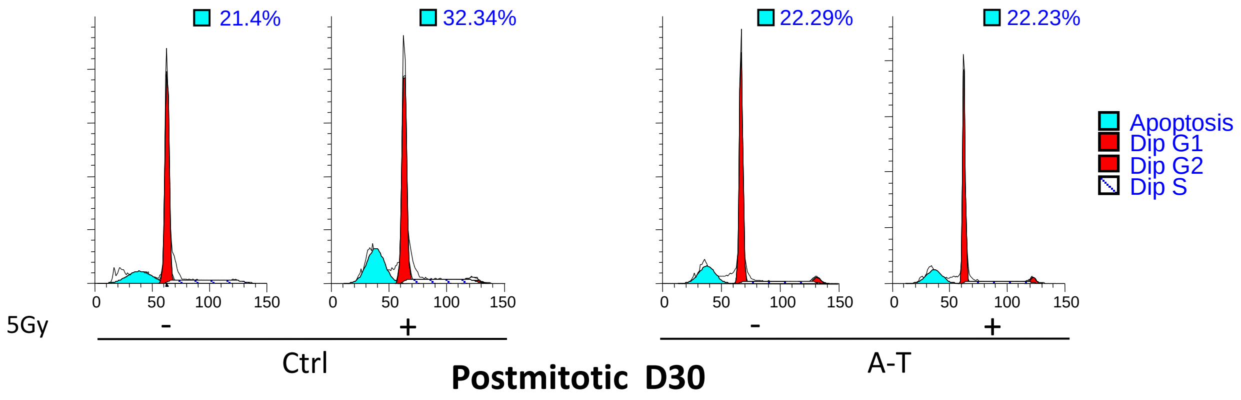

21.4%
32.34%
22.29%
22.23%
0 50 100 150
Apoptosis
Dip G1
Dip G2
Dip S
5Gy
-
+
Ctrl
A-T
Postmitotic D30
